# Innovation in Actinic Keratosis Assessment: Artificial Intelligence-Based Approach to LC-OCT PRO Score Evaluation

**DOI:** 10.3390/cancers15184457

**Published:** 2023-09-07

**Authors:** Fabia Daxenberger, Maximilian Deußing, Quirine Eijkenboom, Charlotte Gust, Janis Thamm, Daniela Hartmann, Lars E. French, Julia Welzel, Sandra Schuh, Elke C. Sattler

**Affiliations:** 1 Department of Dermatology and Allergy, University Hospital, Ludwig Maximilian University of Munich, 80337 Munich, Germanyelke.sattler@med.uni-muenchen.de (E.C.S.); 2 Department of Dermatology and Allergology, University Hospital, University of Augsburg, 86179 Augsburg, Germany; 3 Department of Dermatology & Cutaneous Surgery, Miller School of Medicine, University of Miami, Miami, FL 33136, USA

**Keywords:** actinic keratosis (AK), line-field optical coherence tomography, artificial intelligence, PRO score assessment, non-melanoma skin cancer

## Abstract

**Simple Summary:**

In this study, we evaluated the performance of a previous validated artificial intelligence-based assessment algorithm using line-field confocal optical coherence tomography (LC-OCT) to diagnose actinic keratosis (AK). The AI system accurately graded AK lesions in a large patient cohort and showed high agreement with visual assessments by experts. This non-invasive and fast AI-based approach has the potential to improve the efficiency and accuracy of AK diagnosis, leading to better clinical outcomes for patients.

**Abstract:**

Actinic keratosis (AK) is a common skin cancer in situ that can progress to invasive SCC. Line-field confocal optical coherence tomography (LC-OCT) has emerged as a non-invasive imaging technique that can aid in diagnosis. Recently, machine-learning algorithms have been developed that can automatically assess the PRO score of AKs based on the dermo-epidermal junction’s (DEJ’s) protrusion on LC-OCT images. A dataset of 19.898 LC-OCT images from 80 histologically confirmed AK lesions was used to test the performance of a previous validated artificial intelligence (AI)-based LC-OCT assessment algorithm. AI-based PRO score assessment was compared to the imaging experts’ visual score. Additionally, undulation of the DEJ, the number of protrusions detected within the image, and the maximum depth of the protrusions were computed. Our results show that AI-automated PRO grading is highly comparable to the visual score, with an agreement of 71.3% for the lesions evaluated. Furthermore, this AI-based assessment was significantly faster than the regular visual PRO score assessment. The results confirm our previous findings of the pilot study in a larger cohort that the AI-based grading of LC-OCT images is a reliable and fast tool to optimize the efficiency of visual PRO score grading. This technology has the potential to improve the accuracy and speed of AK diagnosis and may lead to better clinical outcomes for patients.

## 1. Introduction

Actinic keratoses (AKs) are common precancerous skin lesions that develop with chronic exposure to ultraviolet radiation, primarily on sun-exposed areas of the skin [1]. While in AK lesions keratinocyte dysplasia is limited to the epidermis, a loss of the dermo-epidermal- junction (DEJ) can be observed in invasive squamous cell carcinoma (SCC) and defines its invasive proliferation [2]. Therefore, the early detection and appropriate management of AKs is essential to prevent their progression [3].

An important aspect of AK assessment is the use of a clinical scoring system to grade the severity of the lesions [4]. Much like other types of intraepithelial neoplasia, such as vulvar intraepithelial neoplasia (VIN) [5] or anal intraepithelial neoplasia (AIN) [6], Cockerell et al. categorized actinic keratoses (AKs) histologically as keratinocytic intraepithelial neoplasia (KIN) [2]. Another approach was suggested by Röwert-Huber et al. in 2007, where they proposed a purely histological classification. In this system, AKs were divided into three grades (AK I–III) based on the presence of atypical keratinocytes across different layers of the epidermis [7]. Grade I showed atypical keratinocytes in the lower third of the epidermis, grade II in the lower two-thirds, and grade III throughout the entire epidermis. Initially, this classification contributed to the idea of a continuous disease spectrum, suggesting that AKs progress from grade I to grade II and III, ultimately leading to invasive tumor development. However, recent research has revealed that the most prevalent type of AKs associated with invasive squamous cell carcinoma (SCC) is grade I, characterized by atypical keratinocytes exclusively in the basal and suprabasal layers [8]. Consequently, this classification falls short of providing a dependable risk assessment.

To address this limitation and considering that the interaction between the epidermis and dermis, along with tumor invasiveness, originates at the dermoepidermal junction zone, a histomorphological classification based on the basal growth pattern (PRO I–III) of atypical keratinocytes has been proposed [9]. The PRO score is a widely used clinical scoring system for AK-based histological findings at the DEJ [10]. While early-stage PRO I is characterized by clustered and atypical keratinocytes in basal epidermal layers, PRO II shows how small hemispherical buds from the basal epidermis slightly protrude into the upper papillary dermis. In PRO III, we can see spiky or filiform papillary elongations of atypical keratinocytes protrude into the upper dermis [10,11].

Studies indicate that SCC is linked with AKs exhibiting basal proliferation [12]. However, the extent to which the basal growth pattern enables the histomorphological risk stratification of AKs remains to be fully understood. It is crucial to consider that AKs may exhibit histological diversity within a single lesion. Therefore, only the biopsied portion of the tumor can be accurately evaluated. Other parts of the same lesion might display different grades or characteristics.

Although histology continues to be the gold standard, a visual inspection and dermoscopical examination are given preference in the diagnosis and follow-up of AKs due to their non-invasiveness [13]. As a result, and to prevent clinical under- or over-estimation, new emerging imaging techniques have been developed to aid in the management of AKs.

Line-field confocal optical coherence tomography (LC-OCT) is a non-invasive imaging tool providing high-resolution images of tissue microstructures in real-time at depths of up to 500 µm, allowing the visualization of the epidermal-dermal junction at the site of origin of AKs [14,15]. Previous studies have shown that LC-OCT can provide in vivo imaging of AK lesions and allow an evaluation of the downward proliferation pattern of keratinocytes in AK lesions, which is in good agreement with histology [16,17].

In addition to improvements in imaging technology, machine-learning algorithms have been developed that can automatically score skin lesions based on digital images [18,19]. These algorithms use convolutional neural networks (CNNs) [20,21] to identify and classify features in LC-OCT images and can aid in the diagnosis and management of skin lesions. A recent pilot study developed a machine-learning algorithm for the segmentation of skin layers in LC-OCT images to determine automatic PRO score quantification [22].

The aim of this study is to evaluate the performance of a recently developed AI-based PRO scoring algorithm for grading AKs using LC-OCT images in a large cohort of 100 histologically confirmed AK lesions. We hypothesized that AI-based grading would be a more accurate, efficient, and reliable alternative than a visual PRO score assessment.

## 2. Materials and Methods

This retrospective study was conducted on patients with histologically confirmed actinic keratoses who underwent LC-OCT imaging prior to a shave biopsy using the deepLive device (deepLiveTM DAMAE Medical, Paris, France) recruited at the Department of Dermatology and Allergology of the University Hospital at Ludwig Maximilian University (LMU) in Munich and the University Hospital in Augsburg between December 2019 and December 2021. 

A total of 100 lesions were included in this study, comprising 220 acquisitions and a total of 26,674 individual images. For each lesion, two dermatologists and non-invasive imaging experts (J.W., E.S.) reviewed all the LC-OCT acquisitions (single images, videos, and 3D blocks) and selected the most representative one to assess the lesion PRO score. 

Finally, we manually excluded all images with insufficient quality to perform a visual assessment of the PRO score (e.g., lesions presenting thick hyperkeratosis on all images or lesions with inappropriate image acquisition) as well as images with strong artifacts preventing an accurate segmentation of the DEJ, such as air bubbles, black images at the end of the video or 3D images with significant motion artifacts. Selection resulted in a total of 19,898 individual images from 80 lesions, comprising 175 acquisitions with an average of 249 images per lesion used to evaluate the PRO score.

### 2.1. PRO Score Grading System

In concordance with our previous pilot study [22] as well as with Schmitz et al. [23], the PRO score grading system was used to assess the severity of AKs in LC-OCT images. The PRO score is a semi-quantitative scoring system that assigns a numerical value to the thickness and protrusions of the AK lesion at the level of the DEJ [23]. 

Protrusions were detected by comparing the position of the DEJ with a reference estimate of the flat DEJ position. The skin surface was used as an estimate offset by the average thickness of the epidermis. Areas with DEJ below the estimated value were classified as protrusions. Once these regions were identified, the ledge width was quantified as the distance between two intersections where DEJ crossed the estimator.

**Figure 1:**
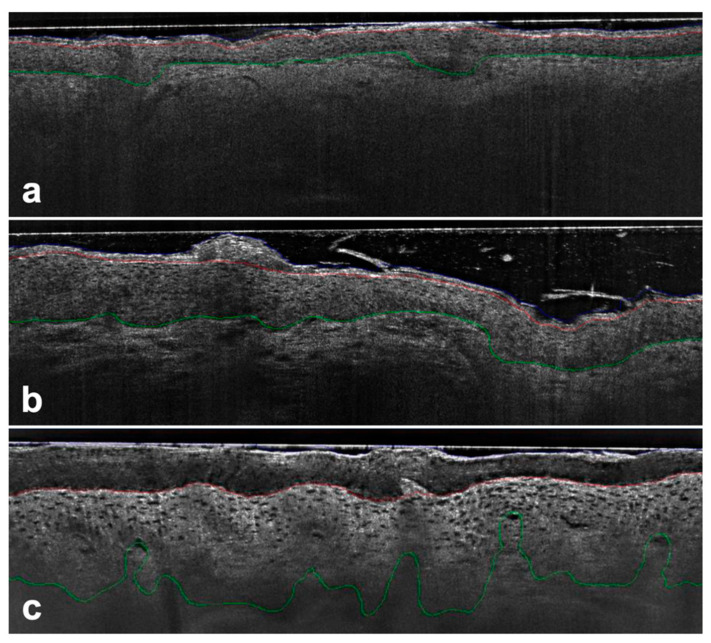
AK PRO score assessment in vertical LC-OCT images (DeepLive, DAMAE Medical, Paris, France) image size: 1.2 × 0.5 mm^2^, lateral and axial resolution: 1.1 μm × 1.3 μm—Protrusions were detected by comparing the position of the DEJ (green line) to a reference estimator in the upper epidermis (red line) with no protrusions detected using the algorithm for PRO I (**a**), while for PRO II (**b**) and PRO III (**c**) the basal epidermis protruded progressively into the dermis.

For each of the 19,898 images analyzed, the undulation of the DEJ, the number of protrusions, and the maximum depth of the protrusion were computed. These metrics were then averaged at the lesion level. The undulation of DEJ was expressed as the length of the DEJ contour divided by the length of the imaged area in a straight line, while undulation depth was calculated as the difference between the highest and lowest points of the DEJ within the interval described by the two intersection points plus a small margin of 10 μm (see Figure 1).

The ground truth for the visual PRO score assessment (PRO I-III) was obtained from the consensus of two dermatologists and noninvasive imaging experts (J.W., E.S.) at LMU und Augsburg University Hospitals. For each lesion, the two experts reviewed all the selected images of the case (single image, video, and 3D) using the review mode of deepLive and reported their estimated average PRO score and maximum PRO score of the lesion.

### 2.2. Machine Learning Assessment

An AI-based algorithm was trained previously on a database of 458 images extracted from 104 AK lesions of 59 patients [22] to automatically score the same set of images for the presence and severity of AKs. This algorithm used a convolutional neural network (CNN) to identify and classify features in the images and generate a PRO score for each lesion. 

This algorithm works on vertical slice images (single images, videos acquired in vertical slice mode, or the vertical projection of 3D stacks) and provides a PRO score for each image. The average lesion PRO score is calculated as the average PRO score from all the images of the lesion. The maximum lesion PRO score is calculated as the maximum PRO score from all the images of the lesion, considering only the maximum score, if present, in at least 10% of the images, to make it more robust to artifacts and outliers.

### 2.3. Statistical Analysis

Descriptive statistics were used to summarize the demographic and clinical characteristics of the study population. The accuracy of the AI-based assessment and the PRO score scoring system was evaluated using sensitivity, specificity, positive predictive value (PPV), and negative predictive value (NPV). The agreement between LC-OCT AI PRO score and the visual expert PRO score was reported as a contingency table, and Cohen’s weighted kappa coefficient with linear weights was used to calculate the correlation between the LC-OCT AI PRO score and visual expert PRO score, including confidence intervals. A *p*-value less than 0.05 was considered statistically significant. 

## 3. Results

A total of 19.898 individual images from 80 lesions were analyzed by AI and were compared to the average visual PRO score per lesion provided by experts.

For the clinical visual average PRO score grading, 21/80 lesions were identified as PRO I, 48/80 as PRO II, and 11/80 as PRO III.

On this dataset, the AI automated average grading per lesion agreed with expert visual grading in 57/80 (71%) of the cases. The weighted kappa for visual and AI classification was κ = 0.51 (*p* < 0.001, CI95% = [0.24, 0.68]). In total, 14/21 (66.7%) were identified successfully as PRO I, 38/48 (79.2%) as PRO II and 5/11 (45.5%) as PRO III. Overall, in 7/80 (9%) of cases, AI overestimated protrusions, while in 16/80 (20%), protrusions were underestimated. 

For PRO I, 7/21 were overestimated as PRO II by AI. In the group of PRO II, 10/48 were underestimated as PRO I, while none were overestimated as PRO III. For PRO III, 6/11 were underestimated as PRO II, but none were underestimated as PRO I. 

Taking the LC-OCT AI maximum PRO score into account, which was calculated as the maximum PRO score from at least 10% of the associated images of one lesion, AI automated grading agreed with the expert visual grading in only 43/80 (54%) of cases. The weighted kappa for the visual and AI maximum PRO score classification was κ = 0.26 (*p* < 0.001, CI95% = [−0.02, 0.35]). In total, 5/21 (23.8%) were identified successfully as PRO I, 31/48 (64.6%) as PRO II and 7/11 (63.6%) as PRO III. Overall, the performances showed a lower correlation than the average grading score (see Table 1).

**Figure 2:**
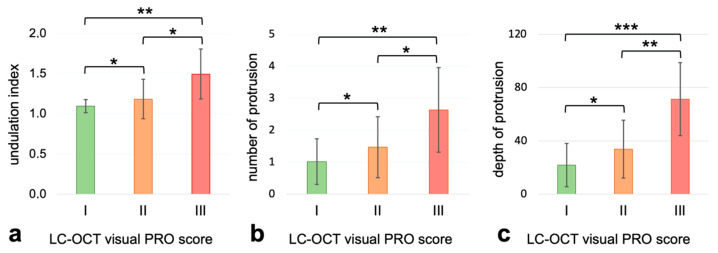
Undulation index, number of protrusions and maximum depth of protrusion in relation to the LC-OCT expert visual PRO score (green: PRO I, orange: PRO II and red: PRO III), * = *p* < 0.05; ** = *p* < 0.005; *** = *p* < 0.001. (**a**) coherent correlation between the visual PRO score and undulation index (ρ: 0.434, CI95% = [0.24, 0.6]), (**b**) the number of protrusions (ρ: 0.434, CI95% = [0.24, 0.6]) and (**c**) the maximum depth of protrusion (ρ: 0.53, CI95% = [0.35, 0.68].

Regarding the undulation index, the number of protrusions, and maximum depth of protrusion, we saw a continuous increase in relation to the growth of the LC-OCT expert visual PRO score. This increase was shown to be statistically significant (*t*-test, *p* < 0.05) and we measured a coherent correlation between the visual PRO score and undulation index (ρ: 0.434, CI95% = [0.24, 0.6]), the number of protrusions (ρ: 0.434, CI95% = [0.24, 0.6]) and the maximum depth of protrusion (ρ: 0.53, CI95% = [0.35, 0.68]) (see Figure 2).

## 4. Discussion

Our present study evaluates the performance of a previously validated artificial intelligence (AI)-based algorithm for the assessment of actinic keratoses (AKs) in comparison to imaging experts’ visual scores in a large cohort of actinic keratoses patients, including a high number of LC-OCT images. 

We demonstrated that compared to the traditional visual PRO score rating, the AI-based assessment shows reliable findings while offering several advantages, including increased objectivity, an improved coverage rate of the lesion, and reduced assessment time.

One of the primary advantages of AI-based assessment is its objectivity [24,25]. Unlike the PRO score rating system, which relies on subjective visual evaluation, the AI algorithm uses a convolutional neural network (CNN) to automatically detect and classify features in the LC-OCT images. This objectivity eliminates interobserver variability, which is often encountered in traditional clinical assessments. Our results demonstrate that the AI-based assessment achieved a high sensitivity of 71.3%. These findings suggest that AI-based assessments can reliably identify AKs, making it a valuable tool for dermatologists in their clinical practice [26,27].

In addition to its objectivity, the AI-based assessment significantly reduces the time required for lesion evaluation. With its availability in live mode during image acquisition at eight frames per second, the AI assessment enables the free scanning of the entire lesion **and** directly obtains the associated AI LC-OCT PRO score from all images without time loss. The ability to quickly assess multiple lesions is particularly crucial in clinical settings with a high caseload, allowing for more efficient patient care and facilitating the early detection of AKs.

Another advantage of the AI-based assessment is its ability to consider the entire lesion in a comprehensive manner. By contrast, the visual rating system relies on individual single images, which might not capture the entire extent of the lesion. In comparison, the AI algorithm analyzes all available images, providing a more comprehensive evaluation of the AK lesion. This broader coverage may likely contribute to a higher sensitivity, as it takes into account the full morphological characteristics of the AK. It is crucial to recognize actinic keratoses as a chronic condition that impacts extensive areas of the skin; thus, treatment objectives should be tailored accordingly. Due to the absence of dependable indicators for predicting the progression of AKs to invasive tumors, rigorous and recurrent treatment, along with preventive measures, are essential approaches to avert any potential advancement to squamous cell carcinoma [28,29]. However, from an economic standpoint and considering the prevalent issue of excessive treatment among patients, establishing an accurate risk stratification becomes imperative [30]. Given the rapid rise in AKs’ incidence, personalized risk assessments, including clinical criteria, the immunocompetence of the patient, as well as the extent of field cancerization, hold the key to ensuring optimal patient care in the future. Drawing from current research, it is conceivable to develop algorithms grounded in specific activating and inhibitory genes to compute an individualized risk score [31,32]. Until this becomes widely feasible, AKs should serve as indicators of chronic photodamage, facilitating early intervention and secondary prevention.

While the PRO score rating system has been widely used as a clinical tool for AK assessment, its classification, in general, does have limitations. The system categorizes AKs into only three stages (PRO I-III), potentially oversimplifying the complexity of these lesions. AKs are known to exhibit a wide range of morphological features, from thin, low-grade lesions to thick, high-grade lesions. The PRO score’s limited staging might not adequately represent the diverse spectrum of AKs, potentially leading to misclassification or underestimation of disease severity.

To address this limitation, a novel approach could be the development of a “Continuous Protrusion Score” (CPS) that offers a more refined and continuous grading system. According to our current results, this CPS should consider multiple factors, such as maximum depth, undulation index, and the number of protrusions, providing a reliable and more accurate representation of AK severity. A CPS could better align with the actual biological continuum of AK progression and may enhance clinicians’ ability to make more precise clinical decisions. Further studies need to investigate which of these factors is the best predictor for AK progression.

## 5. Conclusions

In conclusion, our results highlight, similar to our previous findings, that the additional use of an AI-based assessment tool in LC-OCT images is a reliable and fast means to optimize efficiency and accuracy in the evaluation of actinic keratosis, thus opening new horizons in AK assessment and follow-up treatment. Further studies need to investigate if the assessment of the Continuous Protrusion Score (CPS) can serve as a better predictor for AK progression and may lead to better clinical outcomes for patients.

## Figures and Tables

**Table 1:** Metrics showing congruence between visual PRO score and AI-automated PRO score regarding average score (a) and max 10% score (b) values.

**(a)**				
		**LC-OCT AI PRO score average**		
		I	II	III
**visual** **PRO** **score**	I	14	7	0
	II	10	38	0
	III	0	6	5
**(b)**				
		**LC-OCT AI PRO score max 10%**		
		I	III	III
**visual** **PRO** **score**	I	5	15	1
	II	6	31	11
	III	0	4	7

## Data Availability

The data presented in this study are available on request from the corresponding author. The data are not publicly available due to ethnical and privacy restrictions.
